# Performance of salivary glands ultrasonography, shear wave elastography and their combined use for the diagnosis of primary and secondary Sjögren's syndrome

**DOI:** 10.1002/acm2.14441

**Published:** 2024-07-09

**Authors:** Lian Cheng, Yan Yang, Ai‐Ju Ma

**Affiliations:** ^1^ Department of Ultrasound Medical Imaging Center Affiliated Hospital of Yangzhou University Yangzhou University Yangzhou China; ^2^ Department of Computed Tomography and Magnetic Resonance Imaging Langfang Traditional Chinese Medicine Hospital Langfang Hebei China

**Keywords:** salivary glands, salivary glands ultrasonography, shear wave elastography, shear‐wave velocity, Sjögren's syndrome

## Abstract

**Background:**

Sjögren's syndrome (SS) is a common rheumatic disease for which finding the right imaging tool remains a challenge.

**Purpose:**

This study aimed to evaluate the performance of salivary gland ultrasonography (SGUS), shear wave elastography (SWE) and their combined use for the diagnosis of primary and secondary SS (pSS and sSS).

**Methods:**

This retrospective study included patients with dry symptoms who underwent routine examinations between May 2019 and December 2023. Patients were categorized into the pSS (*n* = 41), sSS (*n* = 26), and control (*n* = 27) groups based on the American College of Rheumatology/European League Against Rheumatism classification criteria (2016). A comparison of SGUS and shear wave velocity (SWV) results was conducted among the three groups. The diagnostic capabilities of different ultrasound methods for SS were evaluated using receiver operating characteristic (ROC) curves and the area under the curve (AUC) for specificity.

**Results:**

Compared to the control group, both the pSS (1.80 ± 1.03 vs. 0.67 ± 0.48, *p* < 0.001) and the sSS (1.85 ± 0.88 vs. 0.67 ± 0.48, *p* < 0.001) groups exhibited significantly elevated SGUS scores. However, there was no statistically significant difference between the pSS and sSS groups (*p* = 0.849). The SWV values in both the pSS and sSS groups were significantly higher than those in the control group (all *p* < 0.001). The AUC for diagnosing SS using only SGUS scores was 0.823 (95% confidence interval [CI]: 0.731–0.894). Combining SGUS scores and SWV values resulted in improved diagnostic accuracy (AUC: 0.883, 95% CI: 0.801–0.940).

**Conclusions:**

SGUS and SWE are pivotal in the diagnosis of Sjögren's syndrome, with their synergistic application poised to bolster diagnostic precision. This combined approach also furnishes substantial backing for the clinical assessment and management of Sjögren's syndrome.

## 1 INTRODUCTION

Primary Sjögren's syndrome (pSS) and secondary SS (sSS) represent a spectrum of autoimmune disorders that primarily affect the salivary and lacrimal glands, culminating in symptoms such as dryness syndrome.^1^ In clinical practice, the precise and rapid diagnosis of pSS and sSS holds paramount significance in formulating effective therapeutic strategies. In recent years, salivary gland ultrasonography (SGUS) and shear wave elastography (SWE), among other imaging modalities, have increasingly attracted attention, offering novel perspectives for the diagnosis of SS.^2^, ^3^

Salivary gland ultrasonography, as a cost‐effective and non‐radiating diagnostic method, has been widely employed for evaluating both the structure and function of salivary glands. Studies have revealed good intra‐ and inter‐observer reliability for SGUS, particularly concerning hypoechoic areas and homogeneity, demonstrating comparable reliability to the histopathological features of SG biopsies in patients with pSS. Nevertheless, the substitution of salivary gland ultrasonography (SGUS) for salivary gland (SG) biopsies markedly diminishes the precision of the classification criteria set forth by the American College of Rheumatology/European League Against Rheumatism (ACR/EULAR, 2016).^4^

Shear wave elastography, a tool for the quantitative assessment of tissue stiffness, is a new and promising diagnostic method for many disorders including SS.^5^, ^6^, ^7^, ^8^, ^9^ As the typical pathological presentation in patients with SS is lymphocytic infiltration and SG fibrosis,^10^ using SWE to assess the stiffness/elasticity of SGs may be helpful in diagnosing SS. Several studies have investigated the diagnostic efficacy of SWE in patients with SS. For example, Świecka et al.^5^ conducted an observational study to detect SWE's effect in diagnosing pSS. The authors found that patients with pSS had significantly higher SWE values for SGs than the controls, demonstrating that SWE could support the diagnosis of pSS. Similarly, Prata et al.^11^ found that total and parotid shear‐wave velocity (SWV), a parameter of SWE, provided good discrimination between patients with pSS and healthy controls.

Although the values of SGUS and SWE in pSS were previously identified, the diagnostic performance of these imaging tools has rarely been explored in sSS. Furthermore, the value of combining SGUS and SWE for an SS diagnosis has not been determined. Thus, this study aimed to evaluate the performance of SGUS and SWE, as well as using them in combination, for the diagnosis of pSS and sSS.

## 2 METHODS

### 2.1 Participants

This study was conducted in accordance with the declaration of Helsinki and approved by the research ethics committee of our hospital (2024‐YKL01‐005). In this study, patients with sicca symptoms, who visited our hospital for routine examinations between May 2019 and December 2023, were screened. All patients were fully assessed for diagnostic approaches concerning SS using the 2016 ACR/EULAR criteria.

Patients who fulfilled the 2016 ACR/EULAR classification criteria and were diagnosed as having pSS were included in the pSS group. Patients who presented with other systemic autoimmune disorders and were diagnosed as having sSS according to the 2016 ACR/EULAR classification criteria were included in the sSS group. Patients with sicca symptoms who did not fulfil the 2016 ACR/EULAR classification criteria for SS were included in the control group. The exclusion criteria included: (1) patients who had previously received head radiotherapy; (2) patients who had undergone surgeries affecting the SGs; (3) patients who had used drugs such as cholinolytics, which can potentially affect salivary secretion; (4) patients with incomplete information about SGUS and SWE; (5) patients younger than 18 years old; (6) patients with a history of hepatitis C infection or acquired immunodeficiency syndrome.

### 2.2 Outcome measures

The clinical data of each patient, including age, gender, and laboratory findings, was retrospectively collected. The data concerning SGUS and SWE were also recorded. The SGUS and SWE for parotid and submandibular glands were performed by two radiologists with more than 5 years of experience using a Siemens ACUSON S3000 ultrasonic diagnostic instrument with a high‐frequency linear array probe, a frequency of 5−12 MHz, and equipped with VTIQ technology software. All patients were placed in the supine position. The parotid glands were thoroughly scanned in both longitudinal and transverse sections along the mandible. The submandibular glands were only scanned in longitudinal sections. The grading system used for diagnosing SS in the present study is presented in Table 1.^12^ The grading scores of four major salivary glands were measured, respectively, and the highest score was recorded as the total score for each patient.

**TABLE 1:** The ultrasonographic grading system for diagnosing Sjögren's syndrome.

Grade	Descriptions
0	Normal parenchyma
1	Mild heterogeneity with hypoechoic regions smaller than 2 mm
2	Marked heterogeneity with hypoechoic regions 2−6 mm
3	Gross heterogeneity with hypoechoic regions greater than 6 mm
4	Adipose degeneration and atrophy

After switching to the shear wave mode, SWE was performed. A region of interest (ROI) was selected on the most representative parts of both the parotid and submandibular glands. Upon transitioning to the standard imaging plane for SWE within the glandular tissue, an ROI measuring 1 × 1 cm was meticulously selected. The transducer was positioned perpendicular to the skin's surface with gentle pressure applied while instructing the patient to relax and momentarily suspend respiration. Following a 3‐s interval to ensure complete color filling within the sampling frame, the acquired frame was stored, and subsequent image processing procedures were diligently executed. Next, SWV (Cs, m/s) was automatically calculated by the software. At least five single measurements were taken for each gland, and the average value was calculated as the final index. The interpretation of SGUS images and SWE measurements was agreed upon by the two radiologists with consensus. If the two radiologists could not reach an agreement, a recommendation from a third radiologist with 15 years of experience was given.

### 2.3 Statistical analysis

Data analysis was conducted using the SPSS 22.0 and MedCalc 20.022 software packages. The presentation of measurement data ensued in the form of mean  ±  SD. The test normal distribution was performed with the Shapiro‐Wilk normality test and the homogeneity of variance was performed with the Levene test. Statistical difference underwent comparison through one‐way ANOVA, succeeded by the Least Significant Difference test. Categorical data were delineated as a percentage (%), with comparisons facilitated by the χ^2^ test or Fisher exact test. The assessment of different ultrasonic methodologies for diagnosing SS was completed by creating receiver operating characteristic (ROC) curves. The area under the curve (AUC) was derived by plotting sensitivity and specificity. The Delong test was used to juxtapose the AUC of the ROC curves. The diagnostic efficacy of combining SGUS and SWE in diagnosing SS was evaluated through logistic regression. A *p*‐value < 0.05 was deemed indicative of statistical significance.

## 3 RESULTS

A total of 94 patients with sicca symptoms (85 women and 9 men) were included in this study. Of the 94 patients, 41 (43.62%, 38 women and 3 men, with a mean age of [56.80 ± 12.54] years) were included in the pSS group; 26 (27.66%) patients with other systemic autoimmune disorders, who were finally diagnosed as having sSS, were included in the sSS group (24 women and 2 men, with a mean age of [44.19 ± 14.68] years). The remaining 27 (28.72%) patients were included in the control group (23 women and 4 men, with a mean age of [57.52 ± 16.95] years). The most common primary systemic autoimmune disorder in the sSS group was rheumatoid arthritis (13/26, 50.00%), followed by systemic lupus erythematosus (7/26, 26.92%), polymyositis (2/26, 7.69%), mixed connective tissue disease (2/26, 7.69%), ankylosing spondylitis (1/26, 3.85%), and ANCA‐associated systemic vasculitis (1/26, 3.85%). Compared with the control group, the pSS and sSS groups had higher rates of anti‐nuclear antibody positivity, anti‐Ro antibody positivity, and anti‐SS‐related antigen A positivity (all *p* < 0.05). Furthermore, the pSS and sSS groups had more patients with a focus score ≥1 compared with the control group (*p* < 0.05). Compared with the pSS group, the sSS group had a higher rate of rheumatoid factor positivity (*p* < 0.05) (Table 2).

**TABLE 2:** Baseline characteristics of the study population (*n* = 94).

Variables	pSS group (*n* = 41)	sSS group (*n* = 26)	Control group (*n* = 27)	*p‐*value
Gender, *n* (%)				0.666
Female	38 (92.68)	24 (92.31)	23 (85.19)	
Male	3 (7.32)	2 (7.69)	4 (14.81)	
Age, years, mean ± SD	56.80 ± 12.54^b^	44.19 ± 14.68	57.52 ± 16.95^b^	0.001
Anti‐nuclear antibody positivity, *n* (%)	30 (73.17)^a^	15 (57.69)^a^	8 (29.63)	0.002
Rheumatoid factor positivity, *n* (%)	9 (21.95)^b^	14 (53.85)	4 (14.81)^b^	0.003
Elevated C reactive protein, *n* (%)	11 (26.83)	6 (23.08)	8 (29.63)	0.864
Low C3 (<76 mg/dL), *n* (%)	12 (29.27)	9 (34.62)	8 (29.63)	0.887
Low C4 (<12 mg/dL), *n* (%)	16 (39.02)	13 (50.00)	6 (22.22)	0.107
High IgG (>16 g/L), *n* (%)	19 (46.34)	16 (61.54)	12 (44.44)	0.380
Anti‐Ro antibody positivity, *n* (%)	23 (56.10)^a^	11 (42.31)^a^	4 (14.81)	0.003
Anti‐Sjögren syndrome‐related antigen A positivity, *n* (%)	17 (41.46)^a^	13 (50.00)^a^	3 (11.11)	0.006
Focus score ≥1, *n* (%)	13 (31.71)^a^	15 (57.69)^a^	0	<0.001

*Note*: Statistic analysis was performed by one‐way ANOVA, χ2 test or Fisher exact test.

^a^ *p* < 0.05 versus control group;.

^b^ *p* < 0.05 versus sSS group.

**FIGURE 1:**
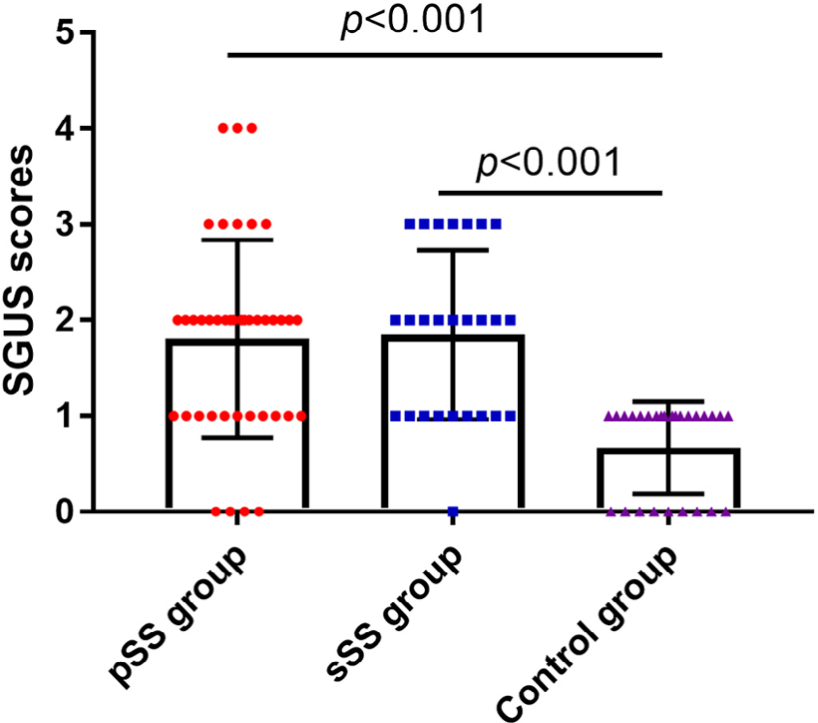
The comparison of salivary gland ultrasonography (SGUS) scores. Statistical analysis was performed by one‐way ANOVA.

The mean SGUS scores in the pSS, sSS, and control groups were 1.80 ± 1.03, 1.85 ± 0.88, and 0.67 ± 0.48, respectively. The SGUS scores were significantly increased in the pSS and sSS groups compared with the control group (all *p* < 0.001, Figure 1). The SGUS score in the pSS group was similar to that in the sSS group (*p* = 0.849, Figure 1).

**FIGURE 2:**
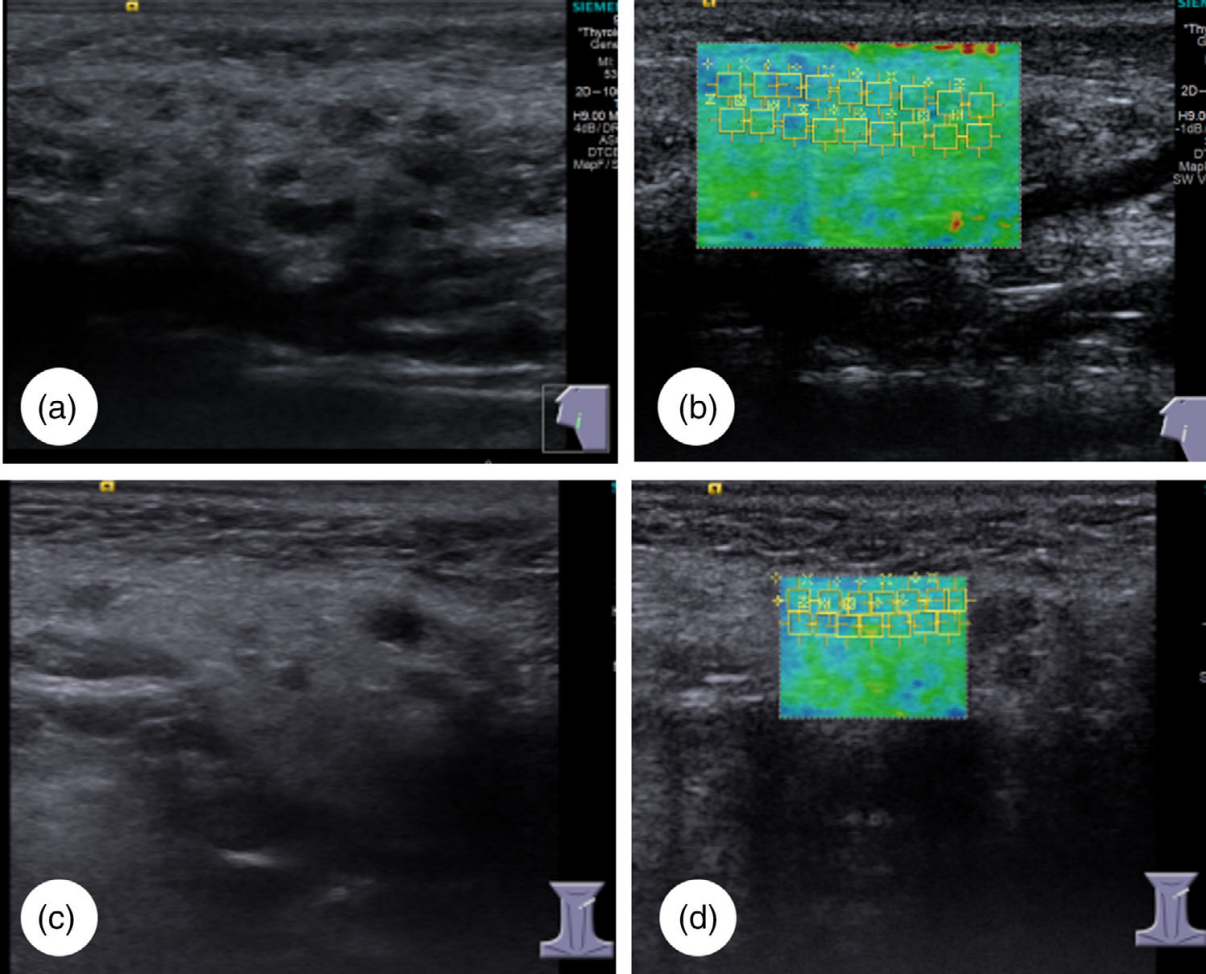
Ultrasonographic features and shear wave elastography of the parotid and submandibular glands in patients with primary Sjögren's Syndrome (pSS). (a) Two‐dimensional ultrasonography of the parotid glands. (b) The shear wave velocity (SWV) of the parotid gland is 2.485 m/s. (c) Submandibular gland two‐dimensional ultrasonography. (d) The SWV of the submandibular gland is 2.36 m/s.

**FIGURE 3:**
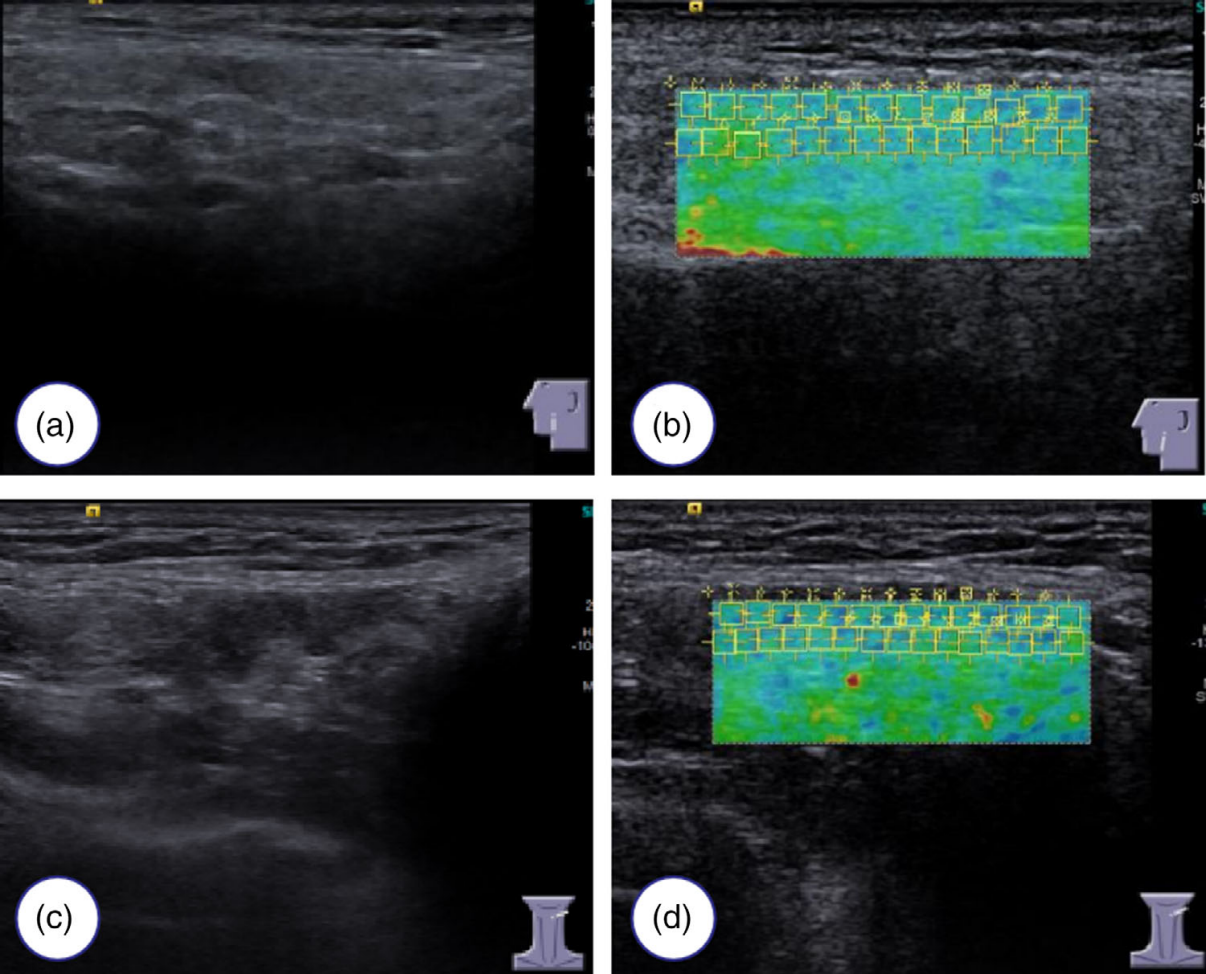
Ultrasonographic features and shear wave elastography of parotid and submandibular glands in patients with in the secondary Sjögren's Syndrome (sSS) Group. (a) Two‐dimensional ultrasonography of the parotid glands. (b) The shear wave velocity (SWV) of the parotid gland is 2.26 m/s. (c) Submandibular gland two‐dimensional ultrasonography. (d) The SWV of the submandibular gland is 2.32 m/s.

**FIGURE 4:**
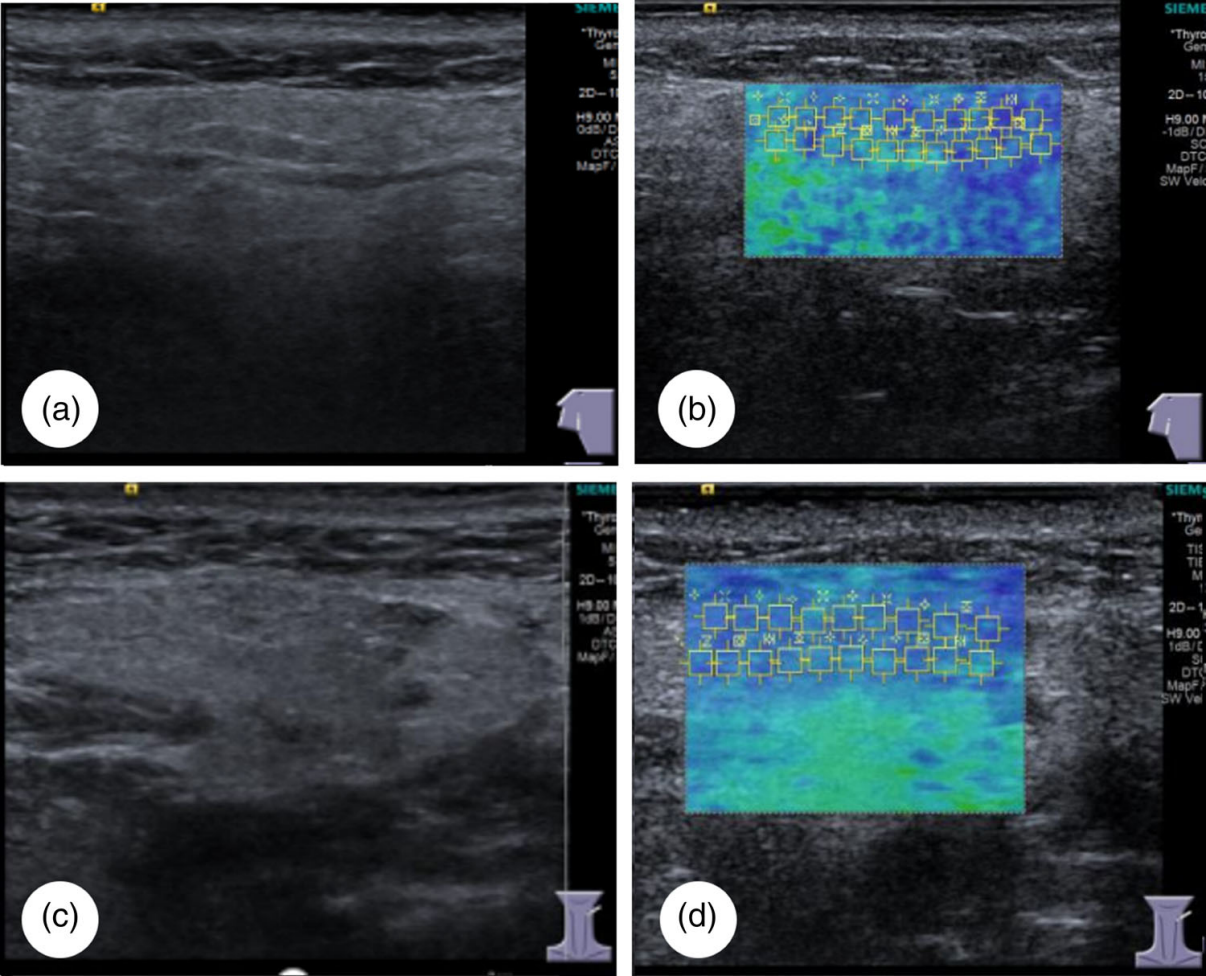
Ultrasound imaging and shear wave elastography of the parotid and submandibular glands in the control group. (a) Two‐dimensional ultrasonography of the parotid glands. (b) The shear wave velocity (SWV) of the parotid gland is 1.699 m/s. (c) Submandibular gland two‐dimensional ultrasonography. (d) The SWV of the submandibular gland is 1.696 m/s.

The SWE results in the study population are presented in Table 3. Patients in the pSS (Figure 2) and sSS groups (Figure 3) had much higher SWV values for the parotid and submandibular salivary glands than those in the control group (Figure 4) (all *p *< 0.05). The SWV values for four salivary glands in the pSS and sSS groups were not significantly different (*p* = 0.908 for the left submandibular gland and *p* = 0.723 for the right submandibular gland; *p* = 0.284 for the left parotid gland and *p* = 0.243 for the right parotid gland, respectively).

**TABLE 3:** Shear‐wave elastography results in the study population (SWV, m/s).

Examined salivary gland	Item	pSS group (*n* = 41)	sSS group (*n* = 26)	Control group (*n* = 27)	*p‐*value
Left submandibular gland	Mean ± SD	2.00 ± 0.40^a^	2.01 ± 0.40^a^	1.59 ± 0.33	<0.001
	Range	1.40–2.81	1.40–2.81	0.83–2.20	
Right submandibular gland	Mean ± SD	2.03 ± 0.38^a^	2.00 ± 0.32^a^	1.61 ± 0.39	<0.001
	Range	1.38–3.30	1.38–2.75	0.82–2.30	
Left parotid gland	Mean ± SD	2.11 ± 0.34^a^	2.21 ± 0.31^a^	1.66 ± 0.51	<0.001
	Range	1.63–2.94	1.71–2.73	0.58–3.00	
Right parotid gland	Mean ± SD	2.10 ± 0.33^a^	2.21 ± 0.37^a^	1.71 ± 0.46	<0.001
	Range	1.59–3.20	1.59–3.20	0.41–2.24	

*Note*: Statistic analysis was performed by one‐way ANOVA.

^a^ *p* < 0.001 versus control group.

**FIGURE 5:**
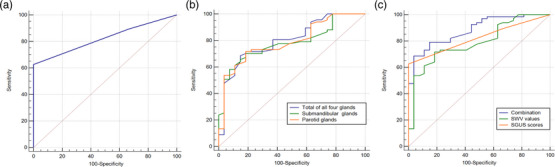
The diagnostic performance in diagnosing Sjögren's syndrome (SS) using different methods. (a) Receiver operating characteristic (ROC) curve of SGUS scores in diagnosing SS. (b) ROC curves of shear‐wave velocity (SWV) values in diagnosing SS. (c) ROC curves of SGUS scores, SWV values, and their combination in diagnosing SS.

Because the pSS and sSS groups had similar SGUS scores and SWV values, we combined the data gained from the pSS and sSS groups to evaluate the diagnostic accuracy of different ultrasonic methods. Figure 5 shows the ROC curves that were constructed by different ultrasonic parameters for diagnosing SS (both pSS and sSS). The SGUS score alone yielded an AUC of 0.823 (95% confidence interval [CI]: 0.731−0.894) for diagnosing SS (Figure 5a). The SGUS score cutoff value was 1, and the corresponding specificity and sensitivity were 100% and 62.69%, respectively. For SWV values, the combined evaluation of both parotid glands and submandibular glands (AUC: 0.802, 95% CI: 0.708−0.877) seemed to have a higher AUC than parotid (AUC: 0.791, 95% CI: 0.695−0.868) or submandibular (AUC: 0.782, 95% CI: 0.685−0.860) (Figure 5b) glands separately. These results indicate that integrating the assessment of these two salivary glands' SWV values may offer enhanced diagnostic accuracy. This could hold significant clinical relevance for early disease detection, disease monitoring, and evaluating treatment efficacy. However, the difference in the AUCs between all four glands and the parotid (*p* = 0.669) or submandibular (*p* = 0.764) glands was not statistically significant. Logistic regression was then used to evaluate the diagnostic performance of combining the SGUS score and SWV value in diagnosing SS (both pSS and sSS). We found that combining these results (AUC: 0.883, 95% CI: 0.801−0.940) had better diagnostic accuracy than using the SGUS score (*p* = 0.036) or SWV value (*p* = 0.006) alone (Figure  5c).

## 4 DISCUSSION

This study aimed to evaluate the efficacy of SGUS score and SWE value, both individually and in combination, in the diagnosis of pSS and sSS. Our findings showed that both the SGUS score and SWE value demonstrated high efficacy in diagnosing pSS and sSS, and combining them further enhanced the diagnostic accuracy. These results present a novel and effective approach for the clinical diagnosis of SS, facilitating early identification and intervention.

Recently, SGUS has gained prominence as a diagnostic tool for SS.^13^, ^14^ It has been well established that SGUS is efficient in terms of discriminating pSS from healthy controls, or sSS from patients with connective tissue diseases.^15^, ^16^, ^17^ However, studies directly comparing SGUS features between pSS and sSS patients are limited. Park et al.^16^ researched the value of SGUS in differentiating sSS from patients with rheumatoid arthritis with sicca symptoms. The researchers found that SGUS scores were much higher in the “rheumatoid arthritis with sSS” group, as well as in the “pSS” group compared with the “rheumatoid arthritis only” group.^16^ In addition, they also found that the “rheumatoid arthritis with sSS” and the “pSS” group had similar SGUS results. Martel et al.^15^ evaluated four SGUS scoring methods for the diagnosis of pSS and sSS. As expected, the scores for all four methods were significantly higher in the pSS and sSS groups than in the control group. However, the diagnostic performance of the four SGUS methods for the diagnosis of pSS and sSS were similar.^15^ Consistent with the above studies, our study obtained a similar conclusion. The SGUS scores showed good diagnostic performance in terms of differentiating a pSS or sSS diagnosis from a control patient with sicca symptoms. However, as pSS and sSS reflected similar ultrasonographic changes, the SGUS scores could not tell them apart.

Shear wave elastography is a promising tool for evaluating tissue stiffness. Recently, the diagnostic value of SWE in the salivary glands of patients with SS has been evaluated in several studies.^12^, ^18^ These studies demonstrated that SWE provided excellent diagnostic performance to discriminate between patients with SS and healthy controls. However, the above studies only focused on pSS. The diagnostic value of SWE in sSS remains unknown. In the present study, as was anticipated, we found that SWE could discriminate a pSS diagnosis from controls. Different from previous studies that included healthy participants as controls, our control group comprised patients with sicca symptoms who did not fulfil the ACR/EULAR criteria.

Shear wave velocity is also a useful parameter for diagnosing sSS. Our results showed that patients in the pSS group had significantly higher SWV values for the parotid and submandibular salivary glands compared with those in the control group. The SWV values obtained by SWE could successfully separate patients with sSS from the controls, demonstrating its promising diagnostic value for sSS.

Given the striking similarity in SGUS scores and SWV values between the pSS and sSS groups, we amalgamated the data from both groups to construct ROC curves, thereby assessing the diagnostic capabilities of various ultrasound methods for SS. Previously, the AUCs of SWV values for diagnosing pSS ranged from 0.80 to 0.81.^11^, ^19^ The AUCs of SGUS scores for diagnosing SS (either pSS or sSS) were reported to range from 0.79 to 0.86.^20^ Our results showed that the SGUS score yielded an AUC of 0.823 (95% CI: 0.731−0.894), and the SWV value yielded an AUC of 0.802 (95% CI: 0.708−0.877) for diagnosing SS. More importantly, we found that combining the SGUS score and SWV value (AUC: 0.883, 95% CI: 0.801−0.940) had better diagnostic accuracy than using either the SGUS score alone or the SWV value only. A study conducted by Prata et al.^11^ obtained a similar conclusion, that is, that combining the SWV value and SGUS score indicates excellent diagnostic performance for a pSS diagnosis. Thus, combining the SGUS score and SWV value for making an SS diagnosis should be used more commonly in clinical practice.

This study employed advanced ultrasound diagnostic techniques, including SGUS and SWE. The former, as a non‐invasive imaging modality, adeptly delineates the structural and morphological changes within salivary glands, thereby aiding in the early detection of SG abnormalities. Conversely, SWE provides insights into the hardness of SG tissues, thereby introducing a novel perspective for diagnosing SG disorders. The synergistic application of these two techniques endows this study with heightened precision and sensitivity in terms of diagnosing SG diseases. Furthermore, this research encompassed multiple salivary glands (including the parotid and submandibular glands) for assessment, thus facilitating a comprehensive understanding of the overall landscape of SG diseases. Through comparative analyses across different salivary glands, this study found variations in disease manifestations, thereby enriching the pool of information available for clinical diagnosis and treatment. It is worth noting that age may also influence the structure and function of SG tissue and, accordingly, affect the imaging results of SGUS and SWE. Salivary gland tissue in elderly patients may exhibit different morphological characteristics due to age‐related changes, which could potentially impact imaging results and diagnostic accuracy.^21^ Therefore, when evaluating the influence of age factors on the characteristics of the study, the age distribution of patients should be considered, and further analysis should be conducted on the clinical features and differences in SGUS and SWE imaging results among patients in different age groups. In specific studies, the impact of age factors on the diagnosis and treatment of SS should be thoroughly considered to enhance diagnostic accuracy and personalized treatment approaches.

The limitations of this study are as follows. First, this study had a relatively small sample size. Second, it was a single‐center study, and all of the included data were retrospectively collected. Third, we only used one SGUS scoring system to diagnose SS. Other SGUS scoring system results are needed to confirm our conclusions. Thus, the usefulness of SGUS and SWE in the diagnosis of SS should be further confirmed in future via large‐scale prospective studies.

## 5 CONCLUSION

This study uncovered that in both patients with pSS and sSS, the SGUS scores were significantly higher than those in the control group, underscoring the pivotal role of SGUS in the diagnosis of SS. SWV measurements effectively differentiated patients with Sjögren's syndrome from healthy individuals. Furthermore, the integration of SGUS and SWE techniques may further enhance diagnostic accuracy and offer fresh diagnostic perspectives for clinical practitioners. These findings collectively indicate the potential utility of SGUS and SWE in the diagnosis of SS.

## AUTHOR CONTRIBUTIONS

*Study design*: Lian Cheng, Yan Yang, and Ai‐Ju Ma. *Data acquisition*: Lian Cheng, Yan Yang, and Ai‐Ju Ma. *Data analysis and interpretation*: Lian Cheng, Yan Yang, and Ai‐Ju Ma. *Manuscript preparation*: Lian Cheng, Yan Yang, and Ai‐Ju Ma. *Critical revision of the manuscript for intellectual content*: Yan Yang. *Manuscript review*: Lian Cheng, Yan Yang, and Ai‐Ju Ma. Obtaining financing: None.

## CONFLICT OF INTEREST STATEMENT

The authors declare no conflicts of interest.

## ETHICS APPROVAL AND CONSENT TO PARTICIPATE

This study was conducted in accordance with the Declaration of Helsinki and approved by the Research Ethics Committee of Affiliated Hospital of Yangzhou University. Due to the nature of retrospective study and anonymized patient's information, informed consent is waived with the approval of Ethics Committee of Affiliated Hospital of Yangzhou University. All methods were carried out in accordance with relevant guidelines and regulations.

## Data Availability

The datasets used and analyzed during the current study are available from the corresponding author on reasonable request.
